# Supplementary material for: Chain flexibility of medicinal lipids determines their selective partitioning into lipid droplets
Source: Nat Commun. 2022 Jun 24;13:3612. doi: 10.1038/s41467-022-31400-6 (PMC9232528; doi:10.1038/s41467-022-31400-6)
Supplement: Supplementary file 1 — Supplementary Information [file 41467_2022_31400_MOESM1_ESM.pdf]

Chain flexibility of medicinal lipids determines their selective partitioning into lipid droplets

Son *et al.*

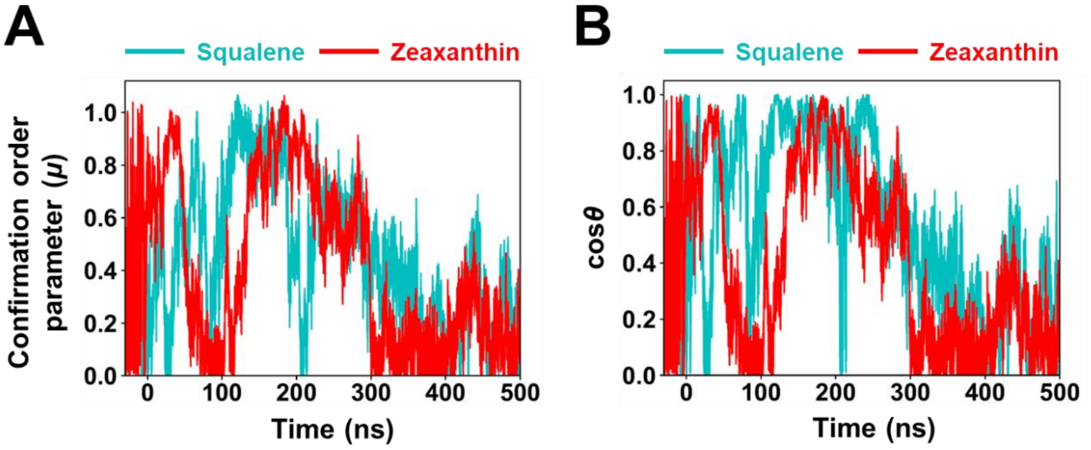

Supplementary Figure 1. Time propagation of conformation order parameter μ in unrestrained MD simulations. (A) Time dependent change of conformation order parameter μ. μ is defined to be $Z_{\text{end-to-end}}/R_{\text{end-to-end}}$, where $Z_{\text{end-to-end}}$ is the z component of $R_{\text{end-to-end}}$, the end-to-end distance of lipids, and this parameter represents the combined effect of the changes in the size ($R_{\text{end-to-end}}$ in **Figure 2D**) and orientation (cosθ in **B**) of the lipid molecules. θ represents the angle between the end-to-end vector of the lipid molecule and the horizontal membrane surface in xy dimension. For flexible squalene, the change in μ can be either correlated (0-200 ns) or independent (200-500 ns) with the lipid size, as shown in **Figure 2D**. For rigid zeaxanthin, μ is independent from $R_{\text{end-to-end}}$, solely representing the change in the orientation of the lipid molecule. **(B)** Time-dependent orientational changes of squalene and zeaxanthin.

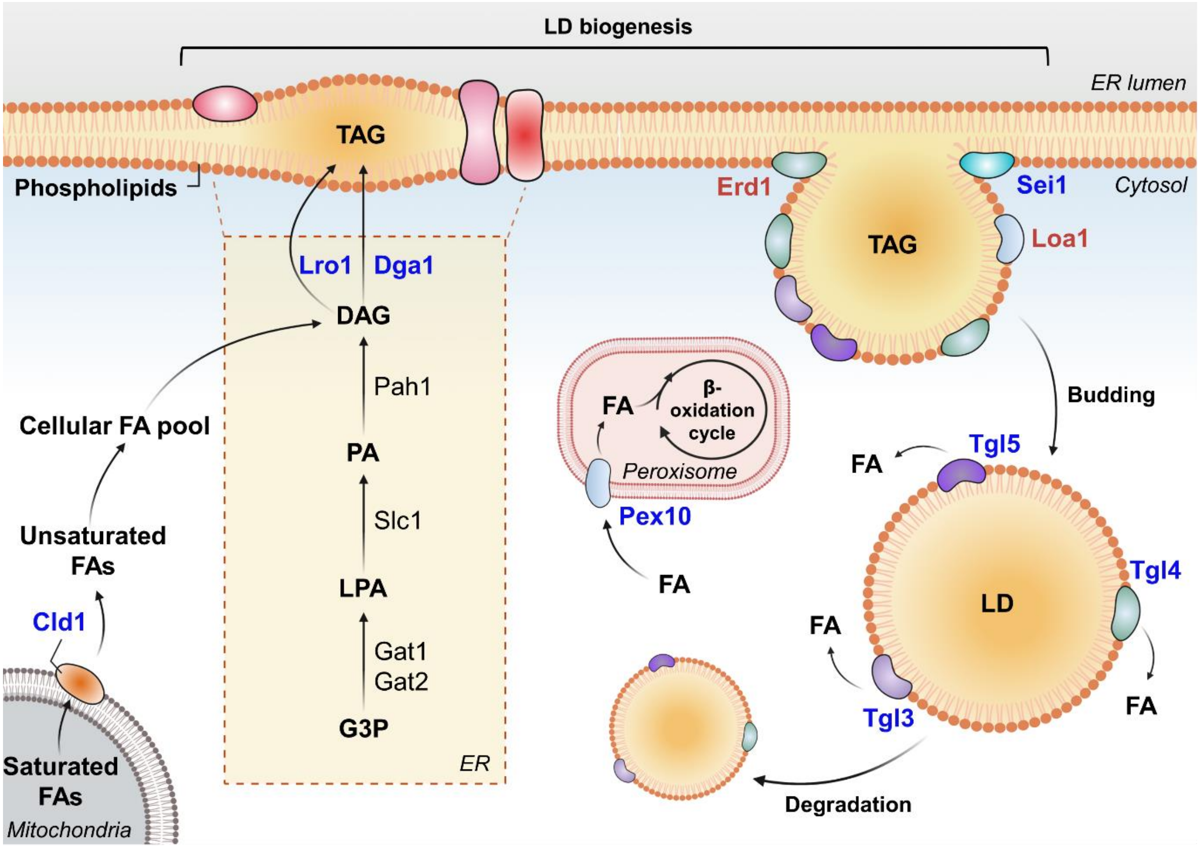

Supplementary Figure 2. Schematic representation of the lipid droplet (LD) biogenesis and degradation. The first step in LD biogenesis is coalescence of neutral lipids such as triacylglycerols (TAGs) and sterol esters (SEs) between the endoplasmic reticulum (ER) bilayer. The TAG synthesis-related proteins, such as Lro1 and Dga1, in the ER membrane devote to the lens formation (see yellow box). Additionally, fatty acids (FAs) which are important sources of neutral lipids can be produced by mitochondrial cardiolipin-specific phospholipase, Cld1. When the concentration of neutral lipids exceeds a certain threshold, LDs bud toward cytosol. The Sei1, Loa1 and Erd1 proteins promote detachment of LDs from ER. The mature LD can be degraded by lipolysis and β-oxidation. The Tgl3, Tgl4, Tgl5, and Pex10 are involved in TAG degradation. The proteins related to increase size and number indicate color-coded in blue and red, respectively.

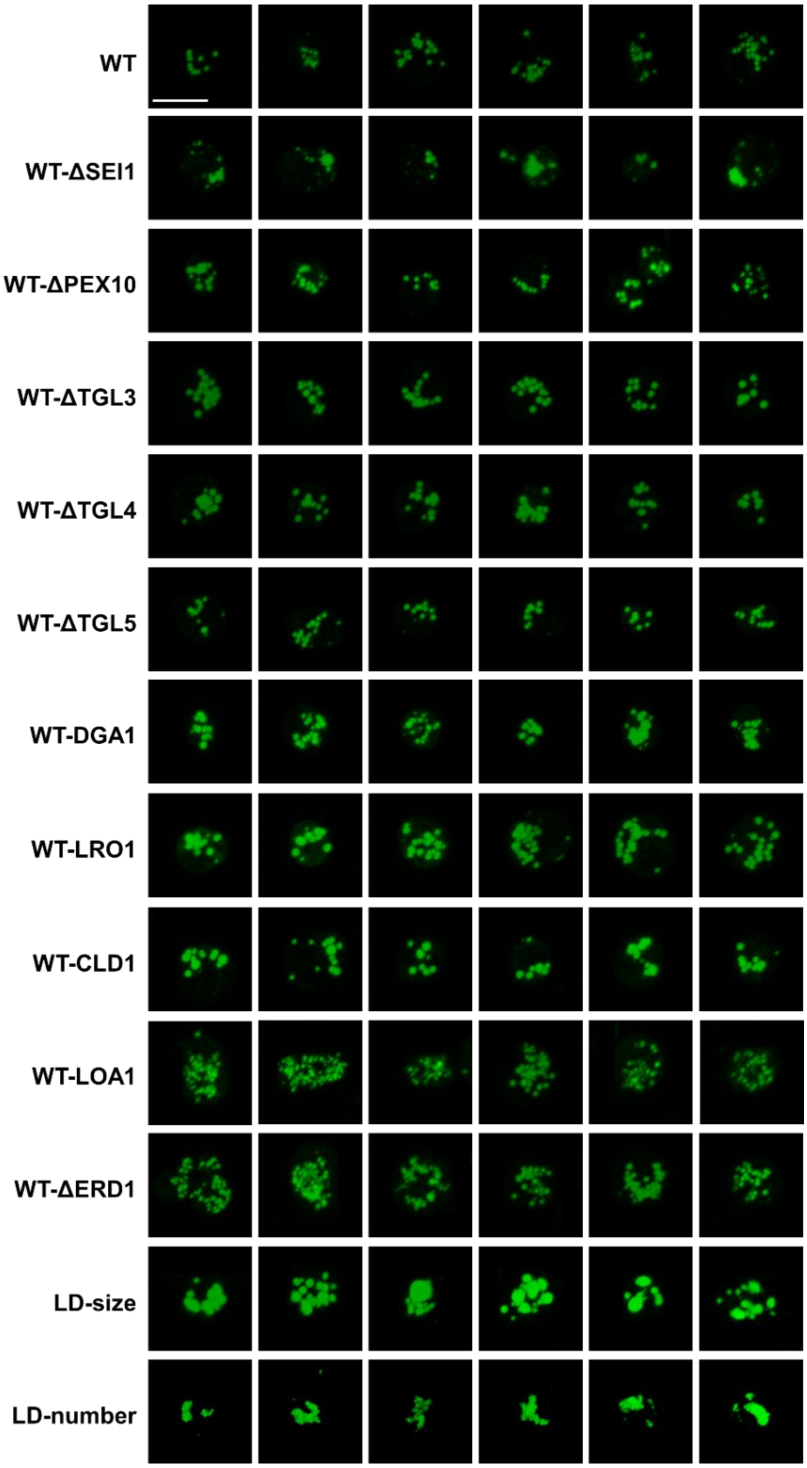

Supplementary Figure 3. Yeast LD remodeling. The cells were grown in YSC medium with 2% (w/v) glucose at 30 °C for 24 h, stained with BODIPY dye, and examined by confocal fluorescence microscopy. The confocal fluorescence microscopy experiments were performed at least triplicate at two independent times.

Scale bar, 5 μm.

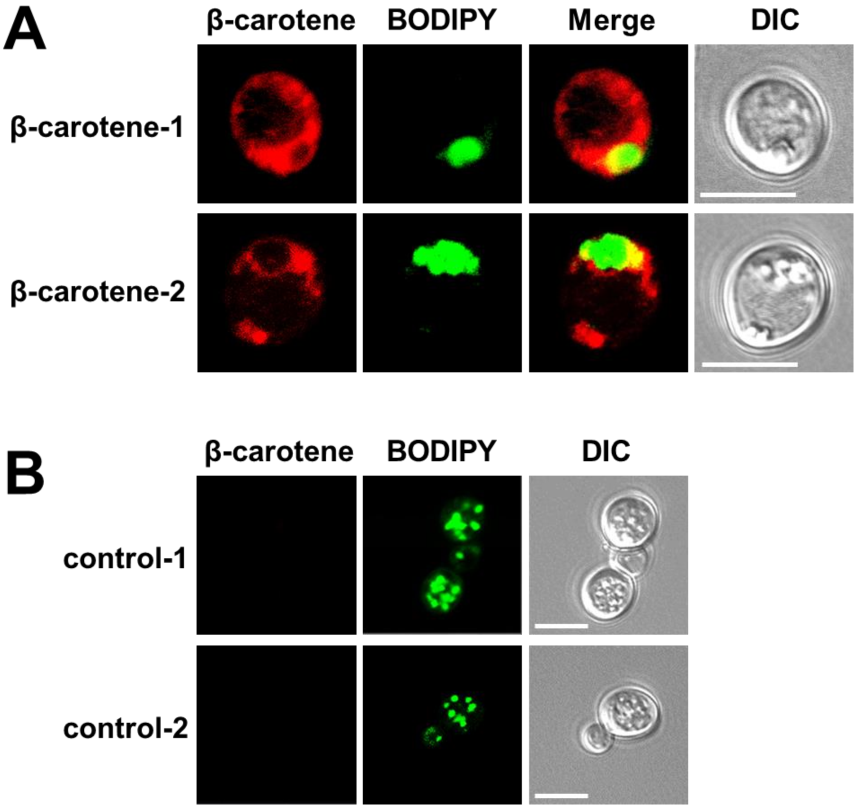

Supplementary Figure 4. β-carotene localization on LD surface. Confocal fluorescence microscopy and differential interference contrast (DIC) images of β-carotene-producing **(A)** and non-producing control cells **(B)**. β-carotene (red) was favorably accommodated on all kinds of membranes including the LD surface (green). The cells were grown in YSC medium with 2% (w/v) glucose at 30 °C. We note that β-carotene was inherently fluorescent (excitation at 450 nm and emission at 600 nm), and LD was stained with the BODIPY fluorescent dye (excitation at 488 nm and emission at 500 nm). The confocal fluorescence microscopy experiments were performed at least triplicate at two independent times. Scale bar, 5 μm.

Supplementary Table 1. Size, number, volume and surface area of LDs in the LD-engineered yeast strains.

	WT	WT-ΔTGL3	WT-LRO1	WT-ΔTGL4	WT-DGA1	WT-CLD1	WT-ΔSEI1	WT-ΔTGL5	WT-ΔPEX10	WT-ΔERD1	WT-LOA1	LD-number	LD-size
**The average diameter of LDs per cell (μm)**	0.200	0.305	0.267	0.305	0.267	0.290	0.314	0.238	0.238	0.207	0.136	0.200	0.330
**The average number of LDs per cell**	23.000	18.583	24.228	15.987	18.344	12.939	8.706	16.467	15.056	31.500	39.500	51.670	15.400
**The total volume of LDs per cell (μm^3)**	0.092	0.279	0.242	0.240	0.183	0.168	0.139	0.115	0.105	0.146	0.051	0.217	0.293
**The total surface area of LDs per cell (μm^2)**	2.898	5.389	5.379	4.636	4.072	3.416	2.699	2.931	2.680	4.240	2.278	6.495	5.267

Supplementary Table 2. Comparison of our LD engineering approach with previous LD oversupply for terpene production

Approaches	Host strain	Target terpene	Fermentation period	Terbinafine (-/+)[a]	Production (mg/g DCW)	Production increase[b]
Our approach	*S. cerevisiae*	Squalene	144 h	-	1.64	**27.3-fold**
				+	5.02	**5.2-fold**
		Zeaxanthin	144 h	-	0.49	**2.5-fold**
				+	0.68	**2.1-fold**
		β-Carotene	144 h	-	3.81	**1.7-fold**
				+	5.66	**1.6-fold**
Previous study[1]	*S. cerevisiae*	Lycopene	96 h	-	70.5	**1.1-fold**
				+	-	**-**

[a] - or + represent the absence or presence of 10 µg/mL of terbinafine in media, respectively.

[b] The ratio of the amount of production by the engineered cells for each approach to that by the non-engineered cells.

Supplementary Note 1. Determination of key genes in creating two different designer yeast cells featuring large LD size and number

Lipid droplets (LDs) are highly dynamic organelles, and alternating between their biogenesis and degradation can cause the dramatic change in LD number and size. The LD biogenesis and degradation are controlled by the enzymes that promote synthesis and hydrolysis of neutral lipids and proteins as involved in budding of LDs from endoplasmic reticulum (ER) (see **Figure 3A** and **Supplementary Figure 2**). Briefly, LD biogenesis starts with the synthesis of neutral lipids by TAG-biosynthetic enzymes, including diacylglycerol (DAG) acyltransferases (Dga1 and Lro1), in the ER membrane. Fatty acids, which are important sources of neutral lipids, can be produced by mitochondrial cardiolipin-specific phospholipase (Cld1), leading to LD formation. Subsequently, LD budding from the ER is facilitated by Seipin (Sei1, formerly known as Fld1), a widely conserved ER membrane protein for normal LD formation at ER-LD contact sites. Loa1 (lysophosphatidic acid acyltransferase) and Erd1 (a predicted membrane protein required for luminal ER protein retention) proteins have also been reported to promote detachment of LDs from the ER to yield mature LDs. The mature LDs can be eventually degraded via lipolysis or lipophagy in response to cellular needs for fatty acids (*e.g.*, nutrient deprivation), and TAG lipases (Tgl3, Tgl4, and Tgl5) hydrolyze the TAGs that are stored in LDs to release fatty acids and other metabolites. In addition, the fatty acids that can be derived from LD lipolysis are broken down for a cellular energy source, and the relevant β-oxidation is performed by peroxisomal membrane E3 ubiquitin ligase (Pex10).

We carefully identified ten different genes of which effects on the LD number or size have been validated by previous studies (**Table 1**), and each gene was deleted or overexpressed in a wild-type (WT) yeast strain, enabling comprehensive evaluation of its specific effect on the LD number and size (see **Figure 3B**, **Supplementary Figure 3**, and **Supplementary Table 1**). Similar with previous studies of LD number and size[2,3] , the cells were grown in YSC medium with 2% (w/v) glucose at 30 °C for 24 h, stained with BODIPY dye (excitation at 488 nm and emission at 500 nm), and examined by confocal fluorescence microscopy.

Approximately, ~300 LDs for every strain were investigated for the statistical analysis of LD number and size.

In terms of increasing the size of LDs, we focused on enhancing neutral lipid accumulation and inhibiting LD budding (**Table 1**), and each mutation of the eight genes yielded a modest increase (1.2- to 1.6-fold) in the LD size compared to no mutation of the WT strain (**Figure 3B** and **Supplementary Table 1**). Specifically, deletion of *sei1* in the WT strain (WT-ΔSEI1) inhibited LD budding, resulting in the highest increase in the LD size (1.6-fold, D_{avg} ~ 0.31 µm), although the LD number was markedly reduced (8.706 LD per cell). We also observed that overexpression of *dga1* or *lro1* (WT-DGA1 or WT-LRO1), encoding major TAG synthases, resulted in an approximately 1.3-fold increase in the LD size (D_{avg} ~ 0.27 µm). As involved in TAG degradation, each deletion of *tgl3, tgl4, tgl5,* or *pex10* increased the LD size (more than 1.2-fold increase, $D_{avg} \geq 0.24$ µm). In addition, to increase cellular fatty acids in providing TAG precursors, diacylglycerols (DAGs), we overexpressed *cld1* in the WT strain. The LD size of the resulting strain (WT-CLD1) increased as well up to 0.29 µm (1.5-fold). Consequently, the increase in LD diameter was in the order of WT-ΔSEI1 (D_{avg} ~ 0.31 µm) > WT-ΔTGL3 = WT-ΔTGL4 (0.30 µm) > WT-CLD1 (0.29 µm) > WT-DGA1 = WT-LRO1 (0.27 µm) > WT-ΔPEX10 = WT-ΔTGL5 (0.24 µm) > WT (0.20 µm) strains (**Figure 3B** and **Supplementary Table 1**).

In terms of increasing the number of LDs, we overexpressed *loa1* or deleted *erd1* in the WT strain, and WT-LOA1 and WT-ΔERD1 strains were generated, respectively. We note that Erd1 has been predicted as a membrane protein for luminal ER protein retention[4] , but several studies reported that deletion of its encoding gene in yeast caused not only an ER stress response, but also the increased number of LDs and content of TAGs[5,6]; despite the pleiotropic effect, there was no other reported phenotypic alterations, which was the reason why we chose the *erd1* gene for further investigation. In this study, we reconfirmed the effect of the *erd1* deletion in yeast, so the LD number of the WT-ΔERD1 strain (31.5 LDs per cell) became 1.4-fold larger than that of the WT strain (**Figure 3B** and **Supplementary Table 1**). Overexpression of *loa1* (39.5 LDs per cell) was even more effective than deletion of *erd1*,

exhibiting an approximately 1.7-fold increase in the LD number, compared to that of the WT strain.

Even though we successfully confirmed the individual gene effect on the LD size or its number in yeast, lots of genes displayed potential trade-off between the size and the number of LDs; for instance, the WT-ΔSEI1 strain was the most effective for increasing the LD size, but its LD number dramatically decreased to be less than 38% of the WT strain's. In this study, we aimed to find the evidence of chain flexibility-dependent lipid migration into LDs: migration of flexible lipids into the LD core and retention of rigid lipids on the LD surface. Hence, instead of including all the mutations, we combined only two or three of the most effective ones to create two different designer yeast cells, LD-size and LD-number, of which regulatory effects can lead to the largest LD volume for confirmation of lipid storage into LDs and the widest LD surface area for observation of lipid retention onto the LDs, respectively.

For the LD-size strain, we combined mutation of three genes (*tgl3*, *tgl4*, and *lro1*) for the largest LD volume (WT-ΔTGL3 ΔTGL4 LRO1), and for the LD-number strain, *loa1* and *erd1* were chosen to induce the widest LD surface (WT-LOA1 ΔERD1). Specifically, in designing the LD-size strain, we observed that among mutations, the order of LD diameter was quite different from that of LD volume; the increase in LD volume was in the order of WT-ΔTGL3 (the total volume of LDs per cell ~ 0.28 μm^3) > WT-LRO1 = WT-ΔTGL4 (0.24 μm^3) > WT-DGA1 (0.18 μm^3) > WT-CLD1 (0.17 μm^3) > WT-ΔSEI1 (0.14 μm^3) > WT-ΔTGL5 (0.12 μm^3) > WT-ΔPEX10 (0.11 μm^3) > WT (0.09 μm^3) strains (**Figure 3B** and **Supplementary Table 1**). In terms of increasing the volume of LDs, deletion of *tgl3* was the most effective, and overexpression of *lro1* and deletion of *tgl4*, both of which similarly affected on the increase of LD volume, was the next most effective. Based on this observation, we combined three mutations to build the LD-size strain (WT-ΔTGL3 ΔTGL4 LRO1). The design of the LD-number strain was simple to be WT-LOA1 ΔERD1 as we evaluated the regulatory effect of only two genes (*loa1* and *erd1*).

Indeed, compared to the WT yeast cells, our engineered LD-size and LD-number cells demonstrated much larger LDs and larger LD population, respectively (**Figure 3C** and

Supplementary Table 1). Although the LD-size cells showed the reduced number of LDs by ~30% compared to the WT cells (23 LDs per cell), the average LD diameter of the LD-size cells increased by ~70% (D_{avg} ~ 0.33 μm), resulting in an ~220% increase in the total LD volume per cell (0.293 μm^3) (**Figure 3C**, middle). We note that the combination of three gene mutations was more effective than each single mutation in increasing the size and volume of LDs (**Supplementary Table 1**). In building LD-number cells, the combination of *loa1* overexpression with *erd1* deletion was highly synergistic. The LD size did not differ between strains (D_{avg} ~ 0.20 μm), but in the LD-number strain, the number of LDs was greater, 51.67 per cell, which was ~130% higher than that of the WT strain, increasing the net surface area of these LDs by ~120% (6.495 μm^2 per cell) (**Figure 3C**, right and **Supplementary Table 1**).

Supplementary references

1 Ma, T. *et al.* Lipid engineering combined with systematic metabolic engineering of *Saccharomyces cerevisiae* for high-yield production of lycopene. *Metab. Eng.* **52**, 134-142 (2019).

2 Wang, C.-W., Miao, Y.-H. & Chang, Y.-S. Control of lipid droplet size in budding yeast requires the collaboration between Fld1 and Ldb16. *J. Cell Sci.* **127**, 1214-1228 (2014).

3 Ayciriex, S. *et al. YPR139c/LOA1* encodes a novel lysophosphatidic acid acyltransferase associated with lipid droplets and involved in TAG homeostasis. *Mol. Biol. Cell.* **23**, 233-246 (2012).

4 Hardwick, K. G., Lewis, M. J., Semenza, J., Dean, N. & Pelham, H. R. ERD1, a yeast gene required for the retention of luminal endoplasmic reticulum proteins, affects glycoprotein processing in the Golgi apparatus. *EMBO J* **9**, 623-630 (1990).

5 Fei, W., Wang, H., Fu, X., Bielby, C. & Yang, H. Conditions of endoplasmic reticulum stress stimulate lipid droplet formation in *Saccharomyces cerevisiae*. *Biochem J* **424**, 61-67 (2009).

6 Teixeira, P. G., David, F., Siewers, V. & Nielsen, J. Engineering lipid droplet assembly mechanisms for improved triacylglycerol accumulation in *Saccharomyces cerevisiae*. *FEMS Yeast Res.* **18** doi: 10.1093/femsyr/foy060 (2018).
