# Supplementary material for: Chain flexibility of medicinal lipids determines their selective partitioning into lipid droplets
Source: Nat Commun. 2022 Jun 24;13:3612. doi: 10.1038/s41467-022-31400-6 (PMC9232528; doi:10.1038/s41467-022-31400-6)
Supplement: Supplementary file 3 — Description of Additional Supplementary Files [file 41467_2022_31400_MOESM3_ESM.pdf]

Description of Additional Supplementary Files

File Name: Supplementary Data 1

Description: Total lipid levels of whole cells and LDs quantified in LD-size and LD-number strains.

File Name: Supplementary Data 2

Description: Comparison of the relative abundance of the different lipid species in LDs of WT, LD-size and LD-number strains.

File Name: Supplementary Data 3

Description: Squalene, zeaxanthin and β-carotene production of LD-engineered strains.

File Name: Supplementary Data 4

Description: List of plasmids and strains used in this study.

File Name: Supplementary Data 5

Description: List of primers in this study.

File Name: Supplementary Movie 1

Description: Deep migration of worm-like squalene into lipid droplets observed by bias-free MD simulations

File Name: Supplementary Movie 2

Description: Surface localization of rod-like zeaxanthin on lipid droplets observed by

bias-free MD simulations
