# Supplementary material for: Chain flexibility of medicinal lipids determines their selective partitioning into lipid droplets
Source: Nat Commun. 2022 Jun 24;13:3612. doi: 10.1038/s41467-022-31400-6 (PMC9232528; doi:10.1038/s41467-022-31400-6)
Supplement: Supplementary file 11 — Reporting Summary [file 41467_2022_31400_MOESM11_ESM.pdf]

nature portfolio

Corresponding author(s): Ju Young Lee, Seung Soo Oh, Chang Yun Son

Last updated by author(s): Jun 8, 2022

Reporting Summary

Nature Portfolio wishes to improve the reproducibility of the work that we publish. This form provides structure for consistency and transparency in reporting. For further information on Nature Portfolio policies, see our Editorial Policies and the Editorial Policy Checklist.

Statistics

For all statistical analyses, confirm that the following items are present in the figure legend, table legend, main text, or Methods section.

n/a	Confirmed	
☐	☒	The exact sample size (*n*) for each experimental group/condition, given as a discrete number and unit of measurement
☐	☒	A statement on whether measurements were taken from distinct samples or whether the same sample was measured repeatedly
☒	☐	The statistical test(s) used AND whether they are one- or two-sided<br>*Only common tests should be described solely by name; describe more complex techniques in the Methods section.*
☒	☐	A description of all covariates tested
☒	☐	A description of any assumptions or corrections, such as tests of normality and adjustment for multiple comparisons
☐	☒	A full description of the statistical parameters including central tendency (e.g. means) or other basic estimates (e.g. regression coefficient) AND variation (e.g. standard deviation) or associated estimates of uncertainty (e.g. confidence intervals)
☐	☒	For null hypothesis testing, the test statistic (e.g. *F*, *t*, *r*) with confidence intervals, effect sizes, degrees of freedom and *P* value noted<br>*Give P values as exact values whenever suitable.*
☒	☐	For Bayesian analysis, information on the choice of priors and Markov chain Monte Carlo settings
☒	☐	For hierarchical and complex designs, identification of the appropriate level for tests and full reporting of outcomes
☒	☐	Estimates of effect sizes (e.g. Cohen's *d*, Pearson's *r*), indicating how they were calculated

Our web collection on statistics for biologists contains articles on many of the points above.

Software and code

Policy information about availability of computer code

Data collection	OpenMM 7.5, CHARMM-GUI, CGenFF
Data analysis	ZEN imaging software 2.1, Image J software 1.8, Visual molecular dynamics version 1.9.4

For manuscripts utilizing custom algorithms or software that are central to the research but not yet described in published literature, software must be made available to editors and reviewers. We strongly encourage code deposition in a community repository (e.g. GitHub). See the Nature Portfolio guidelines for submitting code & software for further information.

Data

Policy information about availability of data

All manuscripts must include a data availability statement. This statement should provide the following information, where applicable:

- Accession codes, unique identifiers, or web links for publicly available datasets
- A description of any restrictions on data availability
- For clinical datasets or third party data, please ensure that the statement adheres to our policy

All data in this study are provided in the Supplementary information/Source Data file. Source data are provided with this paper.

Field-specific reporting

Life sciences study design

All studies must disclose on these points even when the disclosure is negative.

Sample size	Sample size of at least three was chosen following all previous publications in similar field. The sample size is visible in the graphs by individual data points and is stated in each figure legend. Published examples are Braus, S.A.G. et al., Nat Commun. (2022) https://doi.org/10.1038/s41467-022-28690-1 or Makino et al., Nat Commun. (2022) https://doi.org/10.1038/s41467-021-22574-6.
Data exclusions	No data were excluded from analysis.
Replication	All experiments were independently reproduced at least three times. Replicate experiments were successful.
Randomization	Randomization was not applicable in this study because the strains were rationally designed and engineered. Among each engineered strain, the individual colonies were randomly selected.
Blinding	Blinding was not performed for this study because this is not required in this field. We rationally designed and carried out the experiments to achieve our goal, and the data did not require blinding. All data were analyzed and checked by multiple authors and reviewed by the corresponding authors.

Reporting for specific materials, systems and methods

We require information from authors about some types of materials, experimental systems and methods used in many studies. Here, indicate whether each material, system or method listed is relevant to your study. If you are not sure if a list item applies to your research, read the appropriate section before selecting a response.

Materials & experimental systems

n/a	Involved in the study	
☒	☐	Antibodies
☒	☐	Eukaryotic cell lines
☒	☐	Palaeontology and archaeology
☒	☐	Animals and other organisms
☒	☐	Human research participants
☒	☐	Clinical data
☒	☐	Dual use research of concern

Methods

n/a	Involved in the study	
☒	☐	ChIP-seq
☒	☐	Flow cytometry
☒	☐	MRI-based neuroimaging
